# Predicting Oral Beta-lactam susceptibilities against *Streptococcus pneumoniae*

**DOI:** 10.1186/s12879-021-06341-y

**Published:** 2021-07-13

**Authors:** Mark E. Murphy, Eleanor Powell, Joshua Courter, Joel E. Mortensen

**Affiliations:** 1 Division of Infectious Diseases, Cincinnati Children’s Hospital Medical Center, Cincinnati, OH USA; 2 Division of Clinical Pharmacology, Cincinnati Children’s Hospital Medical Center, Cincinnati, OH USA; 3 Department of Pathology and Laboratory Medicine, University of Cincinnati, Cincinnati, OH USA; 4 Division of Pharmacy, Cincinnati Children’s Hospital Medical Center, Cincinnati, OH USA; 5 Division of Pathology, Cincinnati Children’s Hospital Medical Center, Cincinnati, OH USA

**Keywords:** *Streptococcus pneumoniae*, Susceptibility testing; pneumococcus, Beta-lactams (β-lactams), Cefdinir

## Abstract

**Background:**

Oral beta-lactam antimicrobials are not routinely tested against *Streptococcus pneumoniae* due to presumed susceptibility based upon penicillin minimum inhibitory concentration (MIC) testing. Currently, Clinical and Laboratory Standards Institute provides comments to use penicillin MIC ≤0.06 to predict oral cephalosporin susceptibility. However, no guidance is provided when cefotaxime MIC is known, leading to uncertainty with interpretation. The purpose of this study was to evaluate cefotaxime and penicillin MICs and their respective correlation to oral beta-lactam categorical susceptibility patterns.

**Methods:**

249 *S. pneumoniae* isolates were identified by matrix-assisted laser desorption ionization-time of flight mass spectrometry (MALDI-ToF) and then tested by broth microdilution method to penicillin, cefotaxime, amoxicillin, cefdinir, cefpodoxime, and cefuroxime.

**Results:**

Using Clinical and Laboratory Standards Institute (CLSI) non-meningitis breakpoints for cefotaxime, 240/249 isolates were classified as susceptible. Of the cefotaxime susceptible isolates, 23% of the isolates are misrepresented as cefdinir susceptible. Amoxicillin correlated well with penicillin MIC breakpoints with only 1 discordant isolate out of 249.

**Conclusion:**

The correlation between amoxicillin and penicillin creates a very reliable predictor to determine categorical susceptibility. However oral cephalosporins were not well predicted by either penicillin or cefotaxime leading to the possible risk of treatment failures. Caution should be used when transitioning to oral cephalosporins in cefotaxime susceptible isolates, especially with higher cefotaxime MICs.

## Background

The Infectious Diseases Society of America (IDSA) guidelines recommend empiric therapy for hospitalized patients for community-acquired pneumonia or Invasive Pneumococcal Disease (IPD) consisting of an intravenous beta-lactam (β-lactams) such as ampicillin or ceftriaxone depending on local antimicrobial resistance rates [1]. With implementation of Antimicrobial Stewardship Programs in the United States (US), there has been a trend towards early transition to oral antimicrobial therapy once patients have clinically improved [2–5]. In the US, the most commonly used oral beta-lactam agents for this transition, per guideline recommendations, are amoxicillin, amoxicillin-clavulanate, cefdinir, cefuroxime, and cefpodoxime [1, 6].

In spite of these guidelines, not all oral beta-lactams are tested in the laboratory because of the assumption of similar class beta-lactams correlate to other beta-lactams in the same class [7]. For *Streptococcus pneumoniae,* Clinical and Laboratory Standards Institute (CLSI) recommends susceptibility testing in different tier groupings, seen in Table 1, which includes a range of antimicrobials including penicillin, cefotaxime, erythromycin, levofloxacin, and vancomycin [7]. In addition, CLSI’s M100 29th edition has provided susceptible, intermediate and resistant breakpoints of many oral beta-lactams referenced in Table 2. Specifically, a noted comment in Table 2G, comment 5 of M100, references penicillin MIC of ≤0.06 μg/ml to predict susceptibility to not only oral beta-lactams including amoxicillin, cefdinir, cefpodoxime, and cefuroxime, but also cefotaxime and meropenem [7]. The recommendation is based on isolates with ≤0.06 μg/ml do not exhibit any beta-lactam mechanisms for resistance and thus will be susceptible to all other beta lactams. What is not conveyed is predicting susceptibility when penicillin MIC is > 0.06 μg/ml. Furthermore, there is no note or reference made about third generation cephalosporins predicting susceptibility of the oral cephalosporins.

**Table 1:** Suggested Grouping of Antimicrobial Agents That Should be considered for Testing and Reporting of *Streptococcus pneumoniae* by CLSI [7]

Group A: Primary Test and Report	Erythromycin, Penicillin, Trimethoprim-Sulfamethoxazole
Group B: Optional Primary Test, Report Selectively	Ceftriaxone, Cefotaxime, Cefepime, Clindamycin, Doxycycline, Levofloxacin, Meropenem, Vancomycin
Group C: Supplemental, Report Selectively	Amoxicillin, Cefuroxime, Ceftaroline, Chloramphenicol, Ertapenem, Linezolid, Rifampin

**Table 2:** CLSI Breakpoints for Select Penicillin and Cephalosporins [7]

	Interpretative Categories and MIC Breakpoints (μg/ml)		
Antimicrobial Agent	Susceptible	Intermediate	Resistant
Penicillin (meningitis)	≤ 0.06	–	≥ 0.12
Penicillin (non-meningitis)	≤ 2	4	≥ 8
Amoxicillin	≤ 2	4	≥ 8
Cefotaxime (meningitis)	≤ 0.5	1	≥ 2
Cefotaxime (non-meningitis)	≤ 1	2	≥ 4
Cefuroxime (oral)	≤ 1	2	≥ 4
Cefdinir	≤ 0.5	1	≥ 2
Cefpodoxime	≤ 0.5	1	≥ 2

Previous studies have compared *S. pneumoniae* in-vitro MICs of penicillin and oral beta-lactams which demonstrated discordance between penicillin MIC to oral beta-lactams [8–10]. In addition to the discordance, the major commercial automated susceptibility platforms have limited oral agents available for routine susceptibility testing of *S. pneumoniae* [11]. Therefore, the aim of this study was to describe whether penicillin and cefotaxime susceptibility results predict oral beta-lactam susceptibility against *Streptococcus pneumoniae*.

## Methods

Clinical isolates were collected from a large tertiary care pediatric hospital and surrounding outpatient. *S. pneumoniae* isolates were collected in the microbiology laboratory from both sterile, *n* = 110, (blood, cerebral spinal fluid, or urine) and non-sterile specimens, *n* = 77, (bronchiolar lavage, sputum, ear drainage, or sinus) collected from 2014 to 2018. In additional repository collection of invasive isolates, *n* = 62, (blood and CSF) from 2001 to 2008 were also included.

Isolates were identified by colony morphology, MALDI-ToF (Vitek MS, bioMerieux, Leone, France) and using biochemical results on the Vitek 2 (bioMerieux, Lyon, France). Isolates were stored frozen at − 80 °C. then sub-cultured twice on 5% sheep blood agar before testing. Overnight growth of the test isolates was suspended in Mueller Hinton broth to produce a final density of 5 × 10^5^ CFU/ml. Isolates were then inoculated into conventional broth microdilution panels prepared with lysed horse blood and Mueller Hinton broth (ThermoFisher Scientific, Oakwood Village, OH). The panels were incubated overnight at 35 °C in ambient air and were examined macroscopically for evidence of growth. An MIC was defined as the lowest concentration of antimicrobial agent that inhibited growth of the test isolate. *S. pneumoniae* ATCC 49619 was used as a control organism, each day of testing [7].

## Results

The MIC_50_ and MIC_90_ for the 249 isolates and each beta-lactam are listed in Table 3. Overall the majority of the isolates were penicillin and cefotaxime susceptible when using the CLSI non-meningitis breakpoint, at 94 and 96% susceptible, respectively. When using the CLSI meningitis breakpoints, susceptible isolates for penicillin and cefotaxime dropped to 62 and 85%, respectively.

**Table 3:** MIC ranges, MIC50 values, and MIC90 values and Percent Susceptible of 249 *S. pneumoniae* isolates

Drug	MIC Range (μg/ml)	MIC50 (μg/ml)	MIC90 (μg/ml)	Susceptible (%)
Amoxicillin	< 0.12 to 16	0.12	2	93.6
Penicillin	< 0.03 to > 4	0.03	2	94
Cefdinir	< 0.03 to > 4	0.06	4	73.9
Cefpodoxime	< 0.03 to > 4	0.03	2	73.5
Cefotaxime	< 0.03 to > 4	0.03	1	96.4
Cefuroxime	< 0.06 to > 8	0.06	4	74.7

**Fig. 1:**
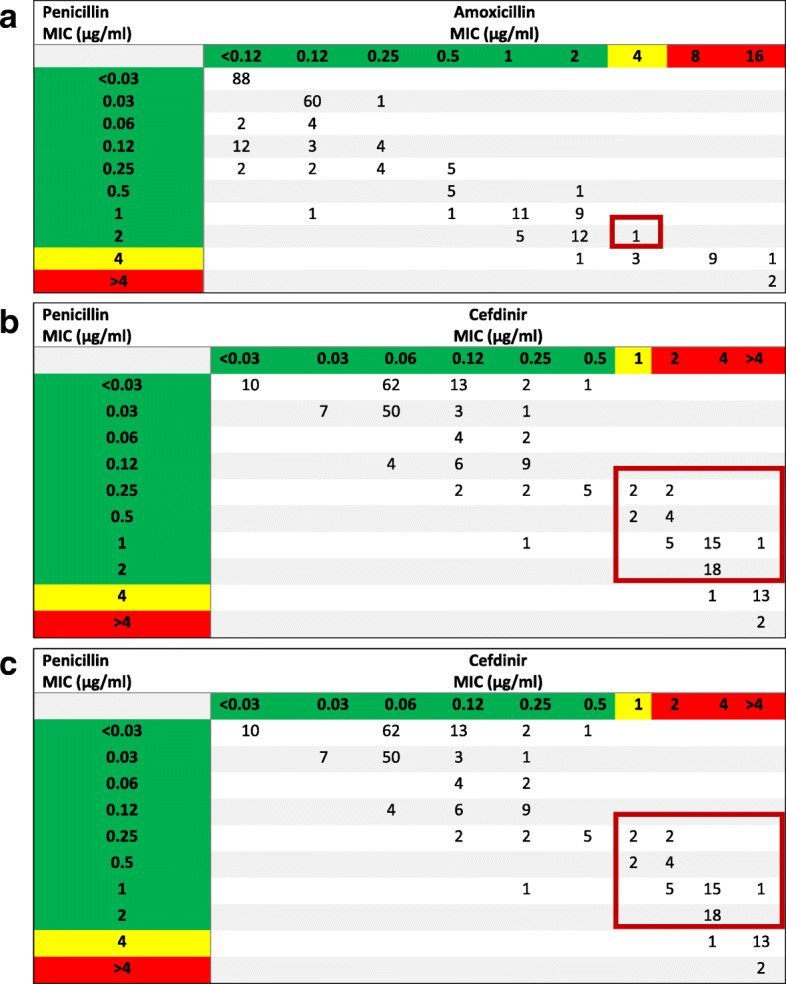
*Streptococcus pneumoniae* isolate MICs (μg/ml) in relationship between penicillin and oral beta-lactams, amoxicillin and cefdinir. **a** and **b**) The correlation between penicillin and oral beta-lactam MICs are represented by amoxicillin and cefdinir. MIC distribution was highlighted to represent CLSI classification of susceptible, intermediate, and resistant MICs using the penicillin non-meningitis breakpoint and respective oral beta-lactam breakpoints. The red box highlights the number of isolates and region for error misclassification. **c**) Use of penicillin MIC ≤0.06 μg/ml to predict cefdinir susceptibility. The red box highlights the discrepancy of the number of isolates that would have been unable to be classified as cefdinir susceptible. **a**) Penicillin and Amoxicillin (249 isolates). **b**) Penicillin and Cefdinir (249 isolates). **c**) Penicillin and Cefdinir with CLSI Guidance (249 isolates)

Penicillin correlated with amoxicillin MICs (Fig. 1A) and using the non-meningitis breakpoint of 2 μg/ml correlated amoxicillin susceptibility at 99%. Cefdinir (Fig. 1B), on the other hand, correlated less with penicillin, with 21% of penicillin-susceptible isolates resistant to cefdinir. When evaluating the CLSI note for Penicillin MIC ≤0.06 μg/ml as a marker for cefdinir susceptibility (Fig. 1C), 18% of cefdinir susceptible isolates are not able to be classified as susceptible. Similar finding were seen with cefpodoxime and cefuroxime.

**Fig. 2:**
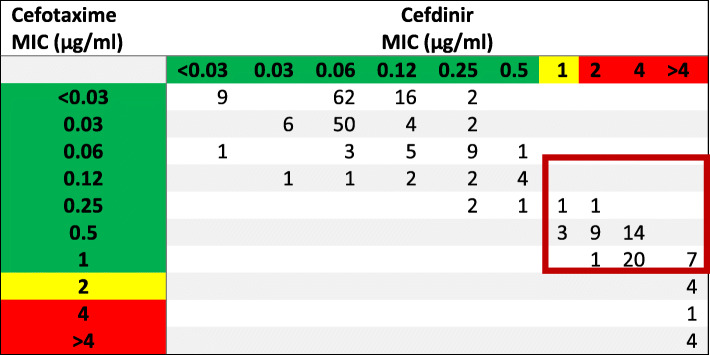
*Streptococcus pneumoniae* isolate MICs (μg/ml) in relationship between cefotaxime and cefdinir. MICs are highlighted in green, yellow, and red to represent susceptible, intermediate and resistant based upon CLSI cefotaxime non-meningitis, and cefdinir breakpoints. Red box annotates the error window of cefotaxime susceptible isolates misclassifying cefdinir resistant isolates

Cefotaxime correlated well with cefdinir susceptibility in the lower range MICs (0.06–0.25), but a larger discordance was noted in the higher cefotaxime MICs (0.5–2) with 23% of the cefotaxime susceptible isolates categorized as cefdinir resistant (Fig. 2). Cefotaxime correlation to cefpodoxime and cefuroxime susceptibility were similar to the cefdinir findings with misrepresentation in 24 and 22% of the isolates respectively.

## Discussion

This study examined the relationship between the susceptibility for cefotaxime and penicillin to predict oral beta-lactams susceptibility. Given the importance of appropriate and timely transition from intravenous to oral therapy, determining the utility of cefotaxime MIC as a reliable marker for oral agents is paramount. However, in this study, the ability of cefotaxime MIC to predict categorical susceptibility of oral cephalosporins was only 77%. Variability in correlating MICs was most notable in the higher range of cefotaxime susceptible MICs (0.25–2 μg/ml). Interestingly, using the CLSI meningitis breakpoint for cefotaxime does have overall good predictability of categorical oral cephalosporins. The use of meningitis breakpoints could be considered as a future marker for oral cephalosporins, but this might cause confusion with clinicians with terms of “meningitis” and oral therapy, as the two are not associated together.

An example of the clinical implications is the most commonly used third-generation oral cephalosporin in the US, cefdinir [12]. In this study, cefdinir had an unfavorably high MIC profile compared to amoxicillin reducing its predictive performance to the commonly tested penicillin or cefotaxime. In addition, cefdinir has poor pharmacokinetic/pharmacodynamic properties, low bioavailability, and short half-life that is unlikely to overcome the higher MIC distribution that was seen in the study [13–15]. Furthermore, there have been limited efficacy and PK studies of cefdinir in the treatment of pneumonia or IPD [16]. These limitations may increase the risk for treatment failure especially in the cefotaxime higher MIC isolates. Similar limitations may apply for the lesser-used cefpodoxime and cefuroxime [17–20].

Using surrogates to infer or predict susceptibility is common in clinical practice with not only *Streptococcus pneumoniae* but essentially any bacterial pathogen that is susceptible to β-lactams [21–24]. It has previously been questioned with other pathogens, notably the Enterobacterales family to cephalosporins. First-generation cephalosporins are similarly used to predict oral cephalosporins against Enterobacterales and has had conflicting results depending on the specific oral cephalosporin tested [22, 23]. With a more recent study proposing routine use of oral cephalosporin, cefpodoxime, susceptibility testing to Enterobacterales as an optimal way to represent susceptibility to other oral cephalosporins (cefdinir, cefixime) [24]. Our data further adds the collection of studies that caution the use of surrogates to represent susceptibility of oral cephalosporins and the first specifically for *Streptococcus pneumoniae*.

CLSI currently only provides interpretation of oral beta-lactam susceptibility when penicillin MIC ≤0.06 μg/ml. What is not offered is when there is mild beta-lactam resistance with a penicillin MIC > 0.06 μg/ml. With antimicrobial resistance rates ever-rising [25] and 37% of our current isolates exhibiting penicillin MIC > 0.06 μg/ml, it is imperative to provide guidance on an efficient way to predict oral beta-lactam susceptibility. Amoxicillin did correlate well with penicillin MIC and would be reasonable to use as a marker for susceptibility. However, in this study, neither penicillin nor cefotaxime was a good predictor for oral cephalosporin susceptibility. If no prediction method can be developed, then routine cefdinir susceptibility may need to be considered as part of standard of care.

There are a few limitations to our study. This was a single center pediatric study which may limit the generalizability to other centers including adult patients that may have different resistant patterns. Additionally, of the sample size studied, most were very penicillin and cefotaxime susceptible limiting the correlation and predictive value of the agents which may wane with higher MIC but susceptible isolates.

## Conclusion

In summary, providers should use caution in assuming oral cephalosporin susceptibility in cefotaxime susceptible isolates. If oral cephalosporin is to be used, specific susceptibility testing should be considered for higher cefotaxime MIC isolates. Alternatively, other agents such as levofloxacin or linezolid could be considered in these situations given the uncertainty of susceptibility and poor PK/PD pharmacokinetics of oral cephalosporins. In addition, CLSI should consider clarification of oral cephalosporin susceptibility when the cefotaxime or ceftriaxone MIC is known.

## Data Availability

The datasets used during the current study are available from the corresponding author on reasonable request.

## Abbreviations

MIC: Minimum inhibition concentration
PK: Pharmacokinetic
PD: Pharmacodynamic
CLSI: Clinical and Laboratory Standards Institute
IPD: Invasive pneumococcal disease
CFU: Colony forming units
MALDI-ToF: Matrix-assisted laser desorption ionization-time of flight mass spectrometry
